# Enhancing Visual-Language Prompt Tuning Through Sparse Knowledge-Guided Context Optimization

**DOI:** 10.3390/e27030301

**Published:** 2025-03-14

**Authors:** Qiangxing Tian, Min Zhang

**Affiliations:** 1 School of Information and Electrical Engineering, Hangzhou City University, Hangzhou 310015, China; 2 School of Computer Science and Technology, East China Normal University, Shanghai 200062, China; zhangmin@westlake.edu.cn

**Keywords:** visual-language models, prompt tuning, sparse knowledge-guided context optimization

## Abstract

Prompt tuning visual-language models (VLMs) for specialized tasks often involves leveraging task-specific textual tokens, which can tailor the pre-existing, broad capabilities of a VLM to more narrowly focused applications. This approach, exemplified by CoOp-based methods, integrates mutable textual tokens with categorical tokens to foster nuanced textual comprehension. Nonetheless, such specialized textual insights often fail to generalize beyond the scope of familiar categories, as they tend to overshadow the versatile, general textual knowledge intrinsic to the model’s wide-ranging applicability. Addressing this base-novel dilemma, we propose the innovative concept of **SparseK**nowledge-**g**uided **Co**ntext **Op**timization (Sparse-KgCoOp). This technique aims to fortify the adaptable prompts’ capacity to generalize to categories yet unencountered. The cornerstone of Sparse-KgCoOp is based on the premise that reducing the differences between adaptive prompt and their hand-crafted counterparts through sparsification operations can mitigate the erosion of fundamental knowledge. Specifically, Sparse-KgCoOp seeks to narrow the gap between the textual embeddings produced by both the dynamic prompts and the manually devised ones, thus preserving the foundational knowledge while maintaining adaptability. Extensive experiments of several benchmarks demonstrate that the proposed Sparse-KgCoOp is an efficient method for prompt tuning.

## 1. Introduction

Leveraging the expansive repository of image–text correlation pairs, the refined visual-language model (VLM) harnesses a broad spectrum of fundamental knowledge, enhancing its capacity to generalize across diverse tasks. The landscape of visual-language modeling has seen a flourishing of innovative approaches like contrastive language-image pre-training (CLIP) [1], Flamingo [2], ALIGN [3], etc. While VLMs are proficient in capturing visual cues and textual interpretations, their refinement is contingent upon a substantial dataset of exceptional quality. Yet, the compilation of such extensive data for model refinement in genuine visual-language scenarios is often impractical. In light of this challenge, the technique of prompt tuning has emerged, offering a means to fine-tune pre-established VLMs for specific downstream applications, and it has demonstrated remarkable efficacy in a plethora of few-shot or zero-shot visual recognition endeavors [4,5,6,7,8,9,10,11].

Visual-language prompt tuning methods, as utilized in this paper, focus solely on textual prompts without delving into visual prompts. This technique typically harnesses task-relevant textual tokens to integrate specific textual knowledge conducive for predictive tasks. In the context of CLIP, the prompt template “a photo of a [Class]” is instrumental in constructing text-based class embeddings for zero-shot predictive tasks [1]. The knowledge encapsulated by these static, crafted prompts—termed general textual knowledge—is known for its broad applicability to novel tasks. However, this knowledge may not sufficiently capture the nuances of downstream tasks as it does not account for each task’s unique requirements. To capture more tailored, task-specific knowledge, approaches like Context Optimization (CoOp) [12] and Conditional Context Optimization (CoCoOp) [13] have been developed, introducing an array of adjustable prompts shaped by labeled samples in few-shot learning settings. The knowledge produced by these adaptable-prompt methods is referred to as specific textual knowledge. However, methods based on CoOp can exhibit limited generalization to unseen categories within the same task, potentially underperforming compared to CLIP for novel classes, as depicted in Table 1.

Given that specific textual knowledge derives from labeled samples in a few-shot setting, it is inherently tuned to recognized classes, which may inadvertently skew it against classes not seen during training, resulting in subpar outcomes in novel domains. For instance, when not trained, CLIP exhibits enhanced accuracy with new, previously unseen classes over methods based on CoOp, as illustrated by respective accuracies: 74.22% for CLIP against 67.99%, and 71.69% for CoOp and CoCoOp. This higher accuracy achieved by CLIP underscores its general textual knowledge’s robustness towards novel classes. Nevertheless, the specialized textual knowledge shaped by CoOp approaches tends to overlook this broad general knowledge, a phenomenon described as catastrophic forgetting. In essence, the more pronounced this forgetting, the more substantial the decline.

In tackling the challenge of preserving general textual knowledge while enhancing the versatility for unseen classes, we propose a pioneering prompt tuning approach, Sparse Knowledge-guided Context Optimization (Sparse-KgCoOp). The crux of Sparse-KgCoOp lies in mitigating the deviation from general textual knowledge, achieved by diminishing the divergence between the learnable and the predefined prompts. Conventionally, the predefined prompts “a photo of a [Class]” are processed by the text encoder of CLIP to procure the general textual embedding, epitomizing the general textual knowledge. On the flip side, Sparse-KgCoOp cultivates a suite of modifiable prompts to yield task-specific textual embedding. Additionally, Sparse-KgCoOp endeavors to shrink the euclidean distance between general and specific textual embeddings, thus retaining crucial general textual knowledge. Echoing the methodology of CoOp and CoCoOp, Sparse-KgCoOp employs the contrastive loss between the task-specific textual and visual embeddings for the refinement of the learnable prompts. Extensive experiments across various experimental settings—including base-to-new generalization, few-shot classification, and domain generalization—demonstrate the effectiveness of Sparse-KgCoOp, spanning 11 image classification datasets. The results, delineated in Table 1, attest to Sparse-KgCoOp’s efficiency: it achieves heightened performance. Our main contributions are summarized as follows:

- We investigate a problem called the base-novel dilemma, i.e., most prompt-tuning visual-language models (e.g., CoOp or CoCoOp) achieve good base-class performance with a sacrifice of new-class accuracy, which is beyond the capability of these methods in real-world data with a mix of base and new classes.
- We propose Sparse-KgCoOp to address the problem. It surpasses existing methodologies in terms of end performance. Notably, it significantly bolsters outcomes for the new class compared to CoOp and CoCoOp, reinforcing the wisdom and indispensability of incorporating general textual knowledge.
- Sparse-KgCoOp’s training expediency aligns with that of CoOp, marking a quicker pace relative to CoCoOp. Results demonstrate the effectiveness of Sparse-KgCoOp.

## 2. Related Work

### 2.1. Vision-Language Models

Advances in recent studies indicate that models synthesizing image–text connections outperform those solely analyzing visual inputs, leading to the creation of potent visual-language models. Such models, rooted in image–text correlations, are referred to as visual-language models (VLMs). Enhancements in VLMs are achieved through several methods: firstly, by deploying more robust textual or visual encoders, such as Transformers [14]; secondly, by applying contrastive representation learning techniques [15]; and thirdly, by integrating an expanded set of imagery [1,3]. Given the dependency of VLM training on extensive labeled datasets, methods like unsupervised or weakly supervised learning are employed to educate the VLMs using images without annotations [16]. Notably, techniques like Masked Language Modeling (MLM) enhance the resilience of visual and textual embeddings by intentionally omitting words from the text [17,18]. Concurrently, Masked Autoencoders [19], which obscure random sections of an input image, have proven to be efficient self-supervised learners. An exemplar of this field is CLIP, which leverages contrastive loss with a visual encoder over an enormous corpus of 400 million image–text pairs, showcasing its adaptability to categories it has not previously encountered. In our approach, we leverage the pre-trained CLIP model, similar to its predecessors CoOp and CoCoOp, to facilitate knowledge transfer. Our proposed method aims to balance the generalization ability of base and novel classes and further improve the performance.

### 2.2. Prompt Tuning

For tailoring pre-trained VLMs to specific downstream applications, prompt tuning methods [1,20] regularly enlist task-specific textual tokens to deduce specialized textual knowledge for the task [1,20]. As an illustration, in CLIP [1], a crafted template like “a photo of a [CLASS]” is instrumental for constructing text embeddings designed for zero-shot predictions. Yet, these fixed prompts might not fully capture the intricacies of downstream tasks due to their generic nature. To surmount this limitation, Context Optimization (CoOp) [12] introduces mutable soft prompts that are shaped using a limited set of annotated samples, thus supplanting the rigid hand-crafted prompts. A notable shortfall of CoOp is that these adaptable prompts remain constant across all images for any given task, thereby disregarding the unique attributes each image may possess. To enhance this, Conditional Context Optimization (CoCoOp) [13] has been introduced, formulating a context tailored to each individual image, and amalgamating it with a context dependent on textual conditions for refining prompt tuning. Notably, it leverages a streamlined neural network to formulate vectors that constitute modifiable textual prompts. ProDA [21] takes this a step further by considering the learning of the prompt’s prior distribution to procure superior task-oriented tokens. Complementarily, ProGrad [22] refines only those prompts whose gradient resonates with the “general knowledge” elicited by the initial prompts. Moreover, DenseCLIP [9] adopts a context-aware strategy for prompt formulation in dense prediction tasks, while CLIP-Adapter [23] incorporates an adapter to fine-tune visual or textual representations. CLIP-Adapter has achieved a significant performance.

Among the current techniques, CoOp and CoCoOp are closely related to our method. CoOp serves as a foundational model for our advanced Sparse-KgCoOp. In contrast to CoOp, Sparse-KgCoOp incorporates a novel element that maintains a minimal divergence between the learnable and original prompts, thereby enhancing its efficacy in classifying novel categories. CoCoOp shares a similar concept with Sparse-KgCoOp in aligning specific learnable knowledge with broader general knowledge. Nevertheless, CoCoOp limits its optimization to aligned prompts and excludes updates that introduce inconsistencies, effectively disregarding substantial conflicting information during the prompt tuning process. In contrast, Sparse-KgCoOp preserves all knowledge, focusing solely on the proximity of specific learnable insights to universal knowledge. Moreover, Sparse-KgCoOp outstrips CoCoOp in terms of efficiency as it eschews the need for supplementary computational efforts. Extensive assessments demonstrate that Sparse-KgCoOp is a proficient technique that achieves superior performance with reduced training durations.

## 3. Methodology

In this paper, we first introduce the contrastive language-image pre-training method (CLIP) and Context Optimization (CoOp). Then, our proposed Sparse Knowledge-guided Context Optimization (Sparse-KgCoOp) is introduced.

### 3.1. Contrastive Language-Image Pre-Training Method

The contrastive language-image pre-training (CLIP) method comprises two encoders—a visual encoder (typically ViT [24] or ResNet [25]) and a text encoder (typically Transformer [14]). The objective of CLIP is to align the embedding spaces of visual and language modalities, and it can be used for zero-shot classification in aligned embedding space. The text is obtained by a predefined template, such as “a photo of a $\left\{class_{i}\right\}$.”, where $\left\{class_{i}\right\}$ represents the *i*-th class name. This input text is then fed into the text encoder to generate ${\left\{w_{i}\right\}}_{i=1}^{K}$, a set of weight vectors, each representing a different category (a total of *K* categories). Simultaneously, image features *x* are generated by the image encoder. Then, similarities between the image vectors and the text vectors are computed, followed by a softmax operation to derive prediction probabilities, which is formulated as

(1) $$p\left(y|x\right)=\frac{\exp(\cos\left(x,w_{y}\right)/\tau )}{\sum_{i=1}^{K}\exp\left(\cos\left(x,w_{i}\right)/\tau \right)},$$

where cos(·,·) denotes cosine similarity and τ is a learned temperature parameter.

### 3.2. Prompt-Based Learning

To enhance the transfer capabilities of the CLIP model and mitigate the need for prompt engineering, the CoOp [12] approach is introduced. CoOp is a prompt tuning technique for vision-language models, which enhances model performance on downstream tasks by optimizing textual prompts. CoOp introduces learnable prompt parameters into pre-trained vision-language models, fine-tuned based on a limited amount of labeled data to adapt to diverse visual understanding tasks. Instead of using “a photo of” as the context, CoOp introduces *M* learnable context vectors, $\left\{v_{1},v_{2},\ldots ,v_{M}\right\}$, each having the same dimension with the word embeddings. The prompt for the *i*-th class, denoted by $T_{i}$:

(2) $$T_{i}=\left\{v_{1},v_{2},\ldots ,v_{M},c_{i}\right\},$$

where $c_{i}$ is the word embedding for the class name. The context vectors are shared among all classes. Let g(·) denote the text encoder, the prediction probability is then

(3) $$p\left(y|x\right)=\frac{\exp(\cos\left(x,g\left(T_{y}\right)\right)/\tau )}{\sum_{i=1}^{K}\exp(\cos\left(x,g\left(T_{i}\right)/\tau \right)},$$

where cos(·,·) denotes cosine similarity and τ is a learned temperature parameter. Notably, the base model of CLIP remains frozen throughout the entire training process.

### 3.3. Sparse Knowledge-Guided Context Optimization

**Figure 1:**
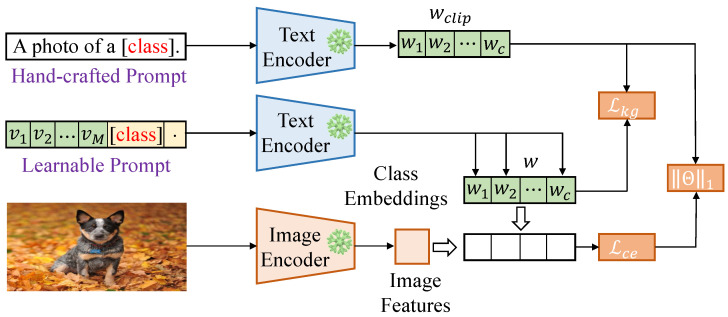
The framework of the Sparse Knowledge-guided Context Optimization for prompt tuning. $L_{ce}$ is the standard cross-entropy loss, and $L_{kg}$ is the proposed Knowledge-guided Context Optimization constraint to minimize the discrepancy between the special knowledge (learnable textual embeddings) and the general knowledge (the textual embeddings generated by the hand-crafted prompt). ${\left||\Theta |\right|}_{\;\;1}$ is the sparse loss, which aims to learn more information.

Building upon CoOp, Sparse Knowledge-guided Context Optimization (Sparse-KgCoOp) incorporates external knowledge, particularly from knowledge graphs, to further guide the generation of textual prompts, as shown in Figure 1. Sparse-KgCoOp leverages the rich structured information within knowledge graphs to enhance the model’s understanding of specific tasks and improve generalization when recognizing unseen categories. It combines information from knowledge graphs with vision-text data to generate richer and more instructive textual prompts, thus improving model performance in the face of diverse data.

Sparse-KgCoOp is based on the assumption that by introducing sparsity in training through feature- and cue-based regularization, we can reduce the model’s overfitting on seen classes while preserving its generalization ability for unseen classes. In the sparse variant of the VLM model, we adjust the embeddings to prioritize key features while reducing the dimensions of our knowledge representation. We denote the sparse textual embedding generated by the CLIP as $w_{\mathrm{clip}}^{\left(i\right)}=\theta \left(t_{\mathrm{clip}}^{\left(i\right)}\right)$ and the refined embedding after sparsification as $w^{\left(i\right)}=\theta_{\mathrm{sparse}}\left(t^{\left(i\right)}\right)$, where $t_{\mathrm{clip}}^{\left(i\right)}$ encapsulates the original vectorized textual tokens, and $t^{\left(i\right)}=\left\{v_{1},v_{2},\ldots ,v_{M},c_{i}\right\}$ represents the learned prompt enriched with sparse representations for the *i*-th class.

The alignment between specialized knowledge, embedded within $w^{\left(i\right)}$, and general knowledge, encapsulated by $w_{\mathrm{clip}}^{\left(i\right)}$, is quantified by the cosine distance, which is a surrogate for the Euclidean distance in a normalized space. This distance, denoted as $L_{kg}$, is inversely proportional to the cosine similarity between the embeddings:

(4) $$L_{kg}=1-\frac{1}{N_{c}}\sum_{i=1}^{N_{c}}\cos\left(w^{\left(i\right)},w_{\mathrm{clip}}^{\left(i\right)}\right)$$

where cos(·,·) represents the cosine similarity, and $N_{c}$ is the number of classes. The score $L_{kg}$ captures the extent to which the specialized knowledge diverges from the general knowledge, with a lower score indicating closer alignment. By minimizing $L_{kg}$, we can effectively enhance the model’s ability to generalize to unseen classes by ensuring that our sparse representations maintain core semantic meanings. Meanwhile, the standard contrastive loss is

(5) $$L_{ce}=-\sum_{x\in X}\log\frac{\exp(d\left(x,w_{y}/\tau \right)}{\sum_{i=1}^{N_{c}}\exp\left(d\left(x,w_{i}\right)/\tau \right)}$$

To enhance the generalization capabilities of our model and to encourage it to focus on the most salient features, we employ a process of sparsification on the learned embeddings. Sparsification is a technique that aims to reduce the complexity of the model by encouraging the majority of the weights to be zero or near zero, effectively reducing the number of active connections within the network. This is achieved by applying a regularization term that penalizes the L1 norm of the weights, inherently promoting sparsity. The refined embedding is thus given by

(6) $$w^{\left(i\right)}=\theta_{\mathrm{sparse}}\left(t^{\left(i\right)}\right)=\mathrm{Sparse}\left(\theta \left(t^{\left(i\right)}\right)\right),$$

where Sparse(·) denotes the sparsification operation applied to the embedding $\theta (t^{\left(i\right)})$.

The overall loss function of our model is a composite of the cross-entropy loss for classification and the sparsification-driven knowledge gap loss $L_{kg}$, which is weighted by a hyperparameter α. Additionally, we incorporate an L1 regularization term scaled by a factor λ to induce sparsity in the parameters. The total loss $L_{\mathrm{total}}$ is thus expressed as

(7) $$L_{\mathrm{total}}=L_{ce}+\alpha \cdot L_{kg}+\lambda \cdot {\left||\Theta |\right|}_{\;\;1},$$

where $L_{ce}$ is the cross-entropy loss, ${\left||\Theta |\right|}_{\;\;1}$ represents the L1 norm of the model parameters, and α and λ are the hyperparameters controlling the strength of the knowledge gap penalty and the sparsity regularization, respectively.

$L_{\mathrm{total}}$ not only ensures proper classification but also drives the model towards a more interpretable and generalizable form by reducing overfitting and promoting the use of fewer, more critical features.

## 4. Experiments

We evaluated the proposed method based on the following settings: (1) generalization from base to new classes within a dataset; (2) few-shot image classification; and (3) domain generalization. All experiments were conducted based on the pre-trained CLIP [1] model.

### 4.1. Experimental Setting

**Dataset.** Following CLIP [1], CoOp [12], and CoCoOp [13], the base-to-new generalization was conducted on 11 image classification datasets, i.e., ImageNet [26] and Caltech [27] for generic object classification; OxfordPets [28], StanfordCars [29], Flowers [30], Food101 [31], and FGVCAircraft [32] for fine-grained visual categorization; EuroSAT [33] for satellite image classification; UCF101 [34] for action recognization; DTD [35] for texture classification; and SUN397 [36] for scene recognition.

**Training Details.** Our implementation was based on CoOp’s codes [12] ( https://github.com/KaiyangZhou/CoOp (accessed on 10 March 2025)) with the CLIP model. We conducted the experiments based on the vision backbone with ResNet-50 [25] and Vit-B/16 [24]. Inspired by CoOp, we fixed the context length to 4 and initialized the context vectors using the template of “a photo of a []”. The final performance was averaged over three random seeds for a fair comparison. We followed the same training epochs, training schedule, and data augmentation setting in CoOp. The hyperparameter λ was set to 8.0. All experiments were conducted based on RTX 3090.

**Baselines.** We considered the baseline models to compare the performance, including CLIP [1], which applies the hand-crafted template “a photo of a []” to generate the prompts for knowledge transfer. CoOp [12] replaces the hand-crafted prompts with a set of learnable prompts inferred by the downstream datasets, which is our baseline. CoCoOp [13] generates the image-conditional prompts by combining the image context of each image and the learnable prompts in CoOp.

### 4.2. Generalization from Base to New Classes

Comparable to the earlier methods, CoOp and CoCoOp, we partitioned each dataset into two segments: foundational classes (base) and emerging classes (new). As shown in Table 2, the proposed Sparse-KgCoOp displayed a higher average performance in terms of harmonic mean (HM) than existing methods on all settings, demonstrating its superiority for the generalization from base to new classes. Specifically, Sparse-KgCoOp outperforms CoOp and CoCoOp in several cases, with the highest HM in Avg dataset, Caltech101, and OxfordPets datasets, where it achieves an HM of 78.25 %, 96.07 %, and 96.90%, respectively. This highlights the robustness of Sparse-KgCoOp when adapting to unseen classes.

Among the existing methods, CoOp obtains the best performance in terms of *base* classes on all settings while obtaining a worse *new* performance than CoCoOp. The reason is that a higher performance on *base* classes makes the CoOp have serious overfitting on the *base* class, thus producing a biased prompt for the *new* classes, leading to a worse *new* performance. Compared with CoCoOp, the proposed Sparse-KgCoOp slightly improves the *base* classes.

### 4.3. Few-Shot Image Classification

**Figure 2:**
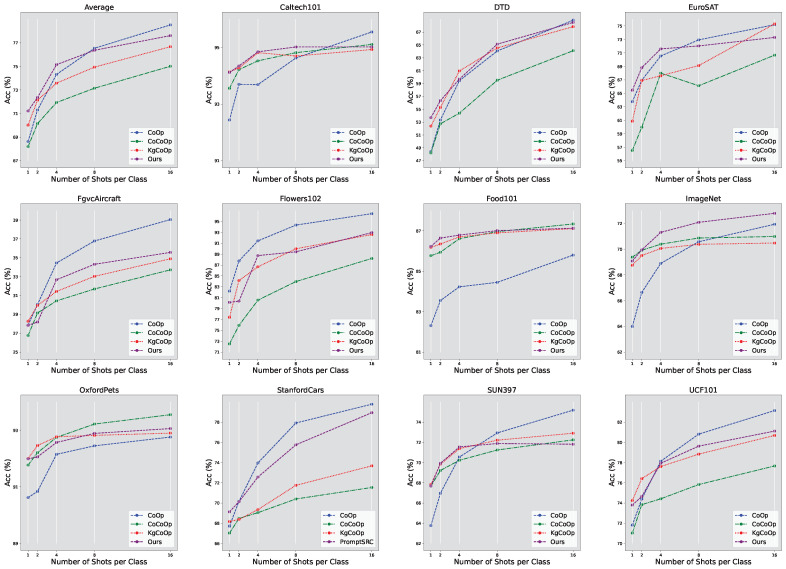
Few-shot learning in image classification on 11 datasets.

The base-to-new configuration posits that the new classes belong to distinct categories compared to the base classes, thereby illustrating the versatility across different categories. To further exhibit the adaptability of our approach, we implemented few-shot classification, training the model with a limited number of labeled images and evaluating it on a dataset comprising the same categories as those used during training. The outcomes from the 4-shot scenario are depicted in Figure 2. It is evident that our developed Sparse-KgCoOp achieves superior average performance relative to existing methods, such as CoOp.

### 4.4. Domain Generalization

Domain generalization seeks to assess generalization by testing the trained model on a target dataset, which shares the same class but exhibits a different data distribution from the source domain. The corresponding outcomes are detailed in Table 3.

From Table 3, it is apparent that CoOp achieves optimal results on the source dataset, ImageNet. This top performance indicates that CoOp can generate a discriminative prompt for the base class, aligning with findings from the base to new scenario. Analogous to the comparison in the base-to-new framework, CoOp displays limited generalization to broader, unseen classes. Among the methods evaluated, CoCoOp demonstrates greater domain generalizability than CoOp. In comparison, our Sparse-KgCoOp surpasses both on the source and target datasets, for instance, elevating the average performance on the target from 62.91% to 66.10%. This comparison underscores that the learnable prompts in Sparse-KgCoOp exhibit enhanced domain generalizability.

## 5. Conclusions

To address the limitations of current CoOp-based prompt tuning methods, which diminish the generalizability for unseen classes, we have developed a prompt tuning strategy called Sparse Knowledge-guided Context Optimization. This method enhances the generalizability of unseen classes by reducing the gap between general textual embeddings and specific learnable textual embeddings. Thorough evaluations across various benchmarks reveal that our proposed Sparse-KgCoOp is a competent prompt tuning approach. While Sparse-KgCoOp enhances generalizability for unseen classes, it might compromise the discriminative capabilities for seen classes; for instance, Sparse-KgCoOp demonstrates suboptimal base performance in seen classes. Future research will focus on developing a balanced approach that effectively addresses both seen and unseen classes.

## Figures and Tables

**Table 1:** Compared to existing methods, the proposed Sparse-KgCoOp is an efficient method, obtaining a higher performance. HM denotes the harmonic mean of base and new class accuracy. All values are reported in percentage (%).

Methods	Prompts	Average over 11 Datasets			Caltech101		
		Base (%)	New (%)	HM (%)	Base (%)	New (%)	HM (%)
CLIP	hand-crafted	69.34	74.22	71.70	96.84	94.00	95.40
CoOp	textual	82.63	67.99	74.60	98.11	93.52	95.76
CoCoOp	textual + visual	80.47	71.69	75.83	97.70	93.20	95.40
**Sparse-KgCoOp (Ours)**	textual	82.75	74.23	**78.25**	97.89	94.32	**96.07**

**Table 2:** Base-to-new generalization performance of baselines and our method on 11 datasets. All values are reported in percentage (%).

Method	Avg over 11 datasets			ImageNet			Caltech101			OxfordPets		
	Base	New	HM	Base	New	HM	Base	New	HM	Base	New	HM
CLIP [1]	69.34	74.22	71.70	72.43	68.14	70.22	96.84	94.00	95.40	91.17	97.26	94.12
CoOp [12]	82.63	67.99	74.60	76.46	66.31	71.02	98.11	93.52	95.76	94.24	96.66	95.43
CoCoOp [13]	80.47	71.69	75.83	75.90	70.73	**73.23**	97.70	93.20	95.40	94.93	97.90	96.39
**Sparse-KgCoOp (Ours)**	82.75	74.23	**78.25**	75.12	70.12	72.53	97.89	94.32	**96.07**	95.56	98.28	**96.90**
Method	StanfodCars			Flowers102			Food101			FGVCAircraft		
	Base	New	HM	Base	New	HM	Base	New	HM	Base	New	HM
CLIP [1]	63.37	74.89	68.65	72.08	77.80	74.83	90.10	91.22	90.66	27.19	36.29	31.09
CoOp [12]	76.20	69.14	72.49	97.63	69.55	81.23	89.44	87.50	88.46	39.24	30.49	34.30
CoCoOp [13]	68.27	70.45	69.34	95.03	69.07	80.00	90.57	91.20	90.88	35.63	32.70	34.10
**Sparse-KgCoOp (Ours)**	77.82	75.01	**76.39**	97.63	73.95	**84.16**	90.67	91.57	**91.12**	38.35	36.20	**37.24**
Method	SUN397			DTD			EuroSAT			UCF101		
	Base	New	H	Base	New	H	Base	New	H	Base	New	H
CLIP [1]	69.36	75.35	72.23	53.24	59.90	56.37	56.48	64.05	60.03	70.53	77.50	73.85
CoOp [12]	80.85	68.34	74.07	80.17	47.54	59.68	91.54	54.44	68.27	85.14	64.47	73.37
CoCoOp [13]	79.50	76.27	77.85	77.37	52.97	62.88	87.97	61.63	72.48	82.33	72.40	77.05
**Sparse-KgCoOp (Ours)**	79.79	76.51	**78.12**	80.05	55.95	**65.86**	93.61	68.61	**79.18**	83.63	75.99	**79.63**

**Table 3:** Domain generalization performance of baselines and our method on 11 datasets. All values are reported in percentage (%).

Method	Source					Target						
	ImgNet	Caltech101	OxfordPets	StanfordCars	Flowers102	Food101	FGVCAircraft	SUN397	DTD	EuroSAT	UCF101	Avg
CoOp [12]	**71.94**	93.39	89.19	62.42	67.1	84.53	18.50	61.44	39.56	39.75	64.22	62.91
CoCoOp [13]	70.99	93.72	90.00	64.98	69.72	86.22	22.90	66.50	44.54	43.08	67.00	65.42
**Sparse-KgCoOp (Ours)**	69.72	**94.44**	**90.44**	**65.56**	**71.17**	**86.33**	**23.62**	**67.08**	**46.32**	**43.54**	**68.90**	**66.10**

## Data Availability

The original contributions presented in this study are included in the article. Further inquiries can be directed to the corresponding author.
